# A Strain-Compensated InGaAs/InGaSb Type-II Superlattice Grown on InAs Substrates for Long-Wavelength Infrared Photodetectors

**DOI:** 10.3390/nano15151143

**Published:** 2025-07-23

**Authors:** Hao Zhou, Chang Liu, Yiqiao Chen

**Affiliations:** 1 Key Laboratory of Artificial Micro- and Nano-Structures of Ministry of Education, School of Physics and Technology, Wuhan University, Wuhan 430072, China; 2017202020055@whu.edu.cn; 2 National Key Laboratory of Materials for Integrated Circuits, Shanghai Institute of Microsystem and Information Technology, Chinese Academy of Sciences, Shanghai 200050, China; 3 Acken Optoelectronics Ltd., Suzhou 215211, China; 4 Center of Materials Science and Optoelectronics Engineering, University of Chinese Academy of Sciences, Beijing 100049, China

**Keywords:** InAs, InGaAs/InGaSb superlattice, infrared detectors, long-wavelength infrared, high quantum efficiency

## Abstract

In this paper, the first demonstration of a highly strained In_0.8_Ga_0.2_As/In_0.2_Ga_0.8_Sb type-II superlattice structure grown on InAs substrates by molecular beam epitaxy (MBE) for long-wavelength infrared detection was reported. Novel methodologies were developed to optimize the As and Sb flux growth conditions. The quality of the epitaxial layer was characterized using multiple analytical techniques, including differential interference contrast microscopy, atomic force microscopy, high-resolution X-ray diffraction, and high-resolution transmission electron microscopy. The high-quality superlattice structure, with a total thickness of 1.5 μm, exhibited exceptional surface morphology with a root-mean-square roughness of 0.141 nm over a 5 × 5 μm^2^ area. Single-element devices with PIN architecture were fabricated and characterized. At 77 K, these devices demonstrated a 50% cutoff wavelength of approximately 12.1 μm. The long-wavelength infrared PIN devices exhibited promising performance metrics, including a dark current density of 7.96 × 10^−2^ A/cm^2^ at −50 mV bias and a high peak responsivity of 4.90 A/W under zero bias conditions, both measured at 77 K. Furthermore, the devices achieved a high peak quantum efficiency of 65% and a specific detectivity (D*) of 2.74 × 10^10^ cm·Hz^1/2^/W at the peak responsivity wavelength of 10.7 µm. These results demonstrate the viability of this material system for long-wavelength infrared detection applications.

## 1. Introduction

Long-wavelength infrared (LWIR) detectors have critical applications across industrial, military, and cosmological domains. Current photodetection technologies include mercury cadmium telluride (MCT) detectors, quantum well infrared photodetectors (QWIPs), and antimonide-based InAs/GaSb superlattice (T2SL) infrared detectors. The past two decades have witnessed remarkable advancements in T2SL infrared detector technology, establishing it as a compelling alternative to traditional MCT detectors [1,2,3,4], with demonstrations of mega-pixel focal plane arrays (FPAs) reaching dimensions of 1K × 1K [5,6,7]. T2SL detectors theoretically offer performances comparable to state-of-the-art MCT detectors due to their larger electron and hole effective masses (resulting in reduced tunneling current) [8] and lower Auger recombination rates [9,10], culminating in superior dark current characteristics. The development of mature GaSb substrates and MBE processes has enabled the realization of high-quality T2SL materials [11,12]. Additionally, the implementation of sophisticated band engineering techniques, leveraging the unique band alignments of InAs, GaSb, and AlSb binary materials, has significantly enhanced T2SL infrared detector performance [4,13,14]. The inherent flexibility of the 6.1 Å family has facilitated the design and growth of innovative barrier structures capable of mitigating generation–recombination (G-R) current in the depletion region, thereby reducing dark currents. Notable advances include M-structure [4], comprising an AlSb/GaSb/InAs/GaSb/AlSb superlattice as an alternative hole-blocking layer design for LWIR detectors. Despite these significant developments, T2SL infrared detectors have yet to achieve their theoretical prediction of a lower dark current compared to HgCdTe [15]. While most research has focused on GaSb-based materials and devices, InAs-based alternatives, especially higher strained structures, remain relatively unexplored. The growth of LWIR InAs/GaSb SL on GaSb substrates requires thick InSb interface layers for strain balancing, presenting significant technical challenges. To address these limitations, we introduce a novel approach utilizing In_0.8_Ga_0.2_As/In_0.2_Ga_0.8_Sb alloys, which exhibit tensile strain (1.34% lattice mismatch) and compressive strain (1.87% lattice mismatch), respectively, relative to InAs substrates. This innovative composition not only resolves interface growth challenges but also exploits strain effects advantageously. The incorporation of Ga provides enhanced flexibility in strain compensation and energy band engineering of strain-compensated In1-xGaxAs/InGa1-ySby SLs, where the interplay of tensile and compressive strains potentially increases valence miniband separation, suppressing Auger recombination. Furthermore, InAs substrates offer superior LWIR transmittance compared to GaSb substrates, particularly beneficial for back-illuminated FPA configurations. In this work, we present the first demonstration of highly strained type-II In_0.8_Ga_0.2_As/In_0.2_Ga_0.8_Sb SL (HS-T2SL) material and photodetector structures grown on InAs substrates by MBE. Following the optimization of InAs buffer and SL growth conditions, PIN structures were fabricated into mesa devices. The comprehensive characterization of surface morphology and structural properties was conducted using differential interference contrast microscopy (DIC), atomic force microscopy (AFM), high-resolution X-ray diffraction (HRXRD), and high-resolution transmission electron microscopy (HRTEM).

## 2. Materials and Methods

**Figure 1:**
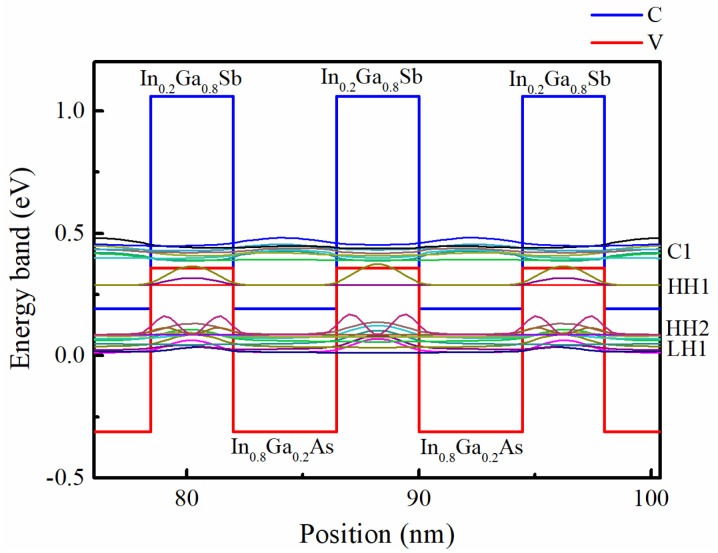
Calculated band structure of In_0.8_Ga_0.2_As (4.45 nm)/In_0.2_Ga_0.8_Sb (3.55 nm) strain-compensated SL along the growth direction with 8 nm period. Conduction miniband (C1), first heavy hole miniband (HH1), second heavy hole miniband (HH2), and first light hole miniband (LH1) are marked in the figure.

The band structure of In_0.8_Ga_0.2_As (4.45 nm)/In_0.2_Ga_0.8_Sb (3.55 nm) strain-compensated SL was calculated by using the 8-band k.p method in the nextnano software (version 3.1.0.0). Band structure simulations at 77 K revealed a bandgap (Ec1-EHH1) of 0.0961 eV, corresponding to a theoretical cutoff wavelength of approximately 12.9 μm. Based on the band structure calculations in Figure 1, the SL samples were engineered to achieve a 100% cutoff wavelength of 13.0 μm, with each period comprising an 8.0 nm total thickness (4.45 nm In_0.8_Ga_0.2_As and 3.55 nm In_0.2_Ga_0.8_Sb sub-layers). The incorporation of 20% Ga into InAs layers and 20% In into GaSb layers generated a tensile strain (−1.34% lattice mismatch) in the In_0.8_Ga_0.2_As sub-layer and a compressive strain (+1.87% lattice mismatch) in the In_0.2_Ga_0.8_Sb sub-layer relative to InAs substrates, resulting in near-zero net strain. The sub-layer thicknesses were maintained below the critical thickness predicted by the Matthews and Blakeslee model [16].

The growth of In_0.8_Ga_0.2_As/In_0.2_Ga_0.8_Sb strain-compensated superlattice (SL) structures was performed using a custom-designed solid-source molecular beam epitaxy system equipped with valved cracker cells for both arsenic and antimony sources. The epitaxial growth was conducted on 2-inch undoped InAs (100) substrates. To achieve the precise control of group III elemental compositions within individual SL layers, the system utilized dual indium and gallium effusion cells. The substrate preparation process followed a rigorous protocol: initial outgassing at 150 °C for 30 min in the loading chamber, followed by secondary outgassing at 300 °C for 30 min in the preparation chamber. Subsequently, the substrates underwent a carefully controlled heating sequence in the growth chamber, initially ramping at 15 °C/min to 500 °C, followed by a more gradual increase at 5 °C/min under As_2_ overpressure until the appearance of a (2 × 4) RHEED reconstruction pattern, marking the deoxidation temperature (Td). The As_2_ overpressure was systematically increased with substrate temperature until reaching 520 °C, corresponding to the maximum pressure required for GaAs growth at approximately 1 μm/h. Thermal oxide desorption was completed at Td + 10 °C for 5 min, culminating in a well-defined (2 × 4) reconstruction RHEED pattern. A critical 100 nm thick InAs buffer layer was deposited at Td −50 °C, maintaining minimal As_2_ flux necessary for stable (2 × 4) surface reconstruction, establishing optimal conditions for subsequent SL growth.

**Figure 2:**
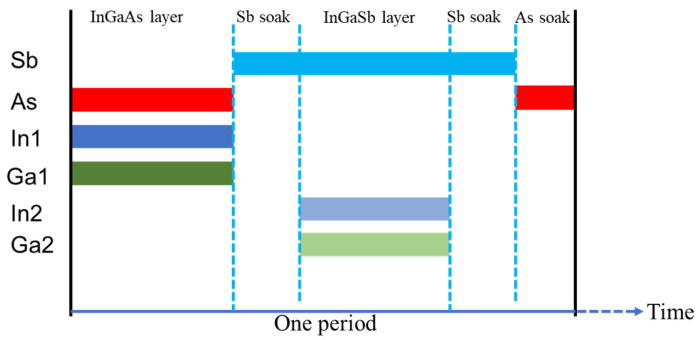
Schematic diagram of shutter sequences for one period of SL.

**Figure 3:**
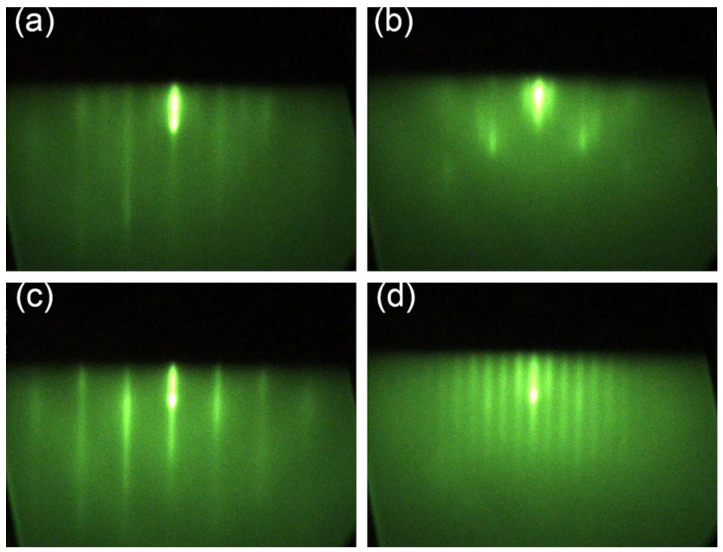
RHEED patterns of (2 × 4) reconstruction for InGaAs constituent layer (**a**,**b**) and (1 × 3) reconstruction for InGaSb constituent layer (**c**,**d**) along [110] azimuth and [1,2,3,4,5,6,7,8,9,10] azimuth, respectively.

Two PIN devices (samples A and B) were grown at temperatures of 400 °C and 385 °C, respectively, for the quality characterization and comparison of materials. The PIN architecture consisted of a 200 nm thick Si-doped N+ contact layer (*n* = 5 × 10^17^ cm^−3^), followed by a non-intentionally doped 1.0 μm absorber region, and a 300 nm thick Be-doped P+ contact layer (*p* = 1 × 10^18^ cm^−3^) with a 3 nm InAs cap layer. The growth rates were precisely controlled at 1 Å/s for sample A and 0.5 Å/s for sample B to maintain optimal material quality at different growth temperatures. Unlike conventional InAs/GaSb T2SL structures, our design eliminated the need for additional strain-balancing interfaces, simplifying the growth process. The growth sequence (Figure 2) incorporated As and Sb soak periods for layer transitions, with initial optimization performed using a 160 nm thick SL under excess As_2_ and Sb_2_ flux conditions to establish correct periodicity and average strain. As2 flux calibration was achieved through surface reconstruction transitions between (2 × 4) and (4 × 2) of the InGaAs layer, utilizing a novel superlattice-based calibration method involving the successive reduction in As_2_ flux during InGaAs/InGaSb SL growth until observing the transition from As-rich 2× to In-rich 4× reconstruction. Sb_2_ flux was calibrated using GaSb homoepitaxy at 1 Å/s. The RHEED analysis shown in Figure 3 revealed clear (2 × 4) patterns for InGaAs sub-layers and (1 × 3) patterns for InGaSb sub-layers, confirming stable growth conditions throughout the epitaxial process.

## 3. Results and Discussion

### 3.1. Materials’ Characterization

**Figure 4:**
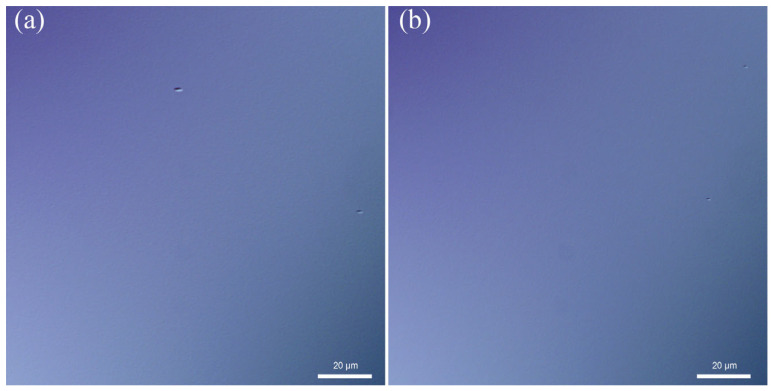
DIC images of (**a**) sample A grown at 400 °C and (**b**) sample B grown at 385 °C. Smooth surfaces with a few defects are observed.

**Figure 5:**
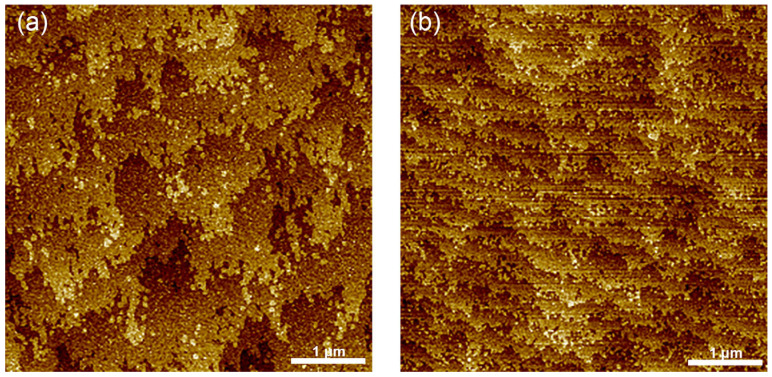
The 5 μm × 5 μm AFM images of (**a**) sample A grown at 400 °C and (**b**) sample B grown at 385 °C. Clear atomic steps can be observed in the graphs. The RMS roughness is (**a**) 0.153 nm and (**b**) 0.141 nm, respectively.

**Figure 6:**
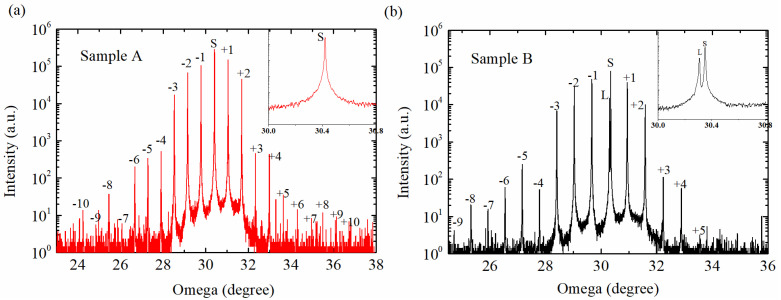
Measured high-resolution X-ray diffraction profiles of the PIN structures. (**a**) Sample A grown at 400 °C; (**b**) sample B grown at 385 °C. The inset shows the zoom-in around the peak of the InAs substrates.

The surface morphology and crystalline quality of the SL epitaxial layers were comprehensively characterized using DIC microscopy, atomic force microscopy, and high-resolution X-ray diffraction. As shown in Figure 4, DIC analysis of both PIN structures (samples A and B) revealed smooth surfaces with minimal defect density. AFM measurements demonstrated exceptional surface quality, exhibiting well-defined atomic steps with root-mean-square (RMS) roughness values of 0.153 nm and 0.141 nm over 5 × 5 μm^2^ areas for samples A and B, respectively, as depicted in Figure 5, confirming the achievement of step-flow growth mode at both growth temperatures. The variation in atomic step density between samples was attributed to subtle differences in the substrate miscut angles. Crystalline quality assessment via HRXRD was performed using a Philips X’pert diffractometer with ω-2θ scan mode for 188-period samples (approximately 1.5 μm thickness). As shown in Figure 6, sample A exhibited up to 10th order satellite peaks with a full width at half maximum (FWHM) value of 15.2 arcsec for the SL +1 satellite peak, while sample B showed 9th order satellite peaks with an FWHM of 18.7 arcsec, both indicating high crystalline quality. The absence of peak splitting in higher-intensity, lower-order peaks further confirmed the stability and precision of the growth conditions.

**Figure 7:**
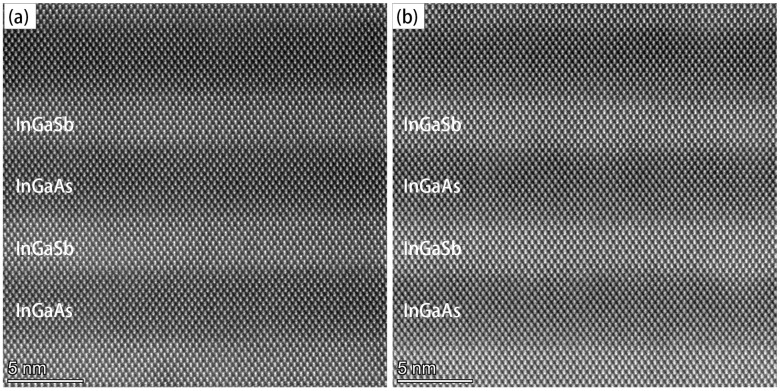
Cross-sectional HAADF-STEM images of PIN structure, taken from absorbing layer of (**a**) sample A grown at 400 °C and (**b**) sample B grown at 385 °C.

High-resolution transmission electron microscopy (HRTEM) analysis was conducted on [110] oriented cross-sectional specimens prepared from samples A and B using conventional mechanical polishing techniques. High-angle annular dark-field scanning transmission electron microscopy (HAADF-STEM) micrographs revealed well-defined periodic sub-layer structures along the growth direction in both samples, as shown in Figure 7. The HAADF intensity contrast, which scales with the square of atomic number (Z), enabled clear differentiation between constituent elements (Sb: Z = 51, Ga: Z = 31, As: Z = 33, In: Z = 49), with InGaSb sub-layers appearing brighter due to the higher Z-value of Sb atoms. Sample A, grown at a higher temperature, exhibited superior interfacial flatness compared to sample B, which showed notable fluctuations at the InGaAs/InGaSb interfaces. This enhanced interface quality was attributed to improved atomic migration kinetics at elevated growth temperatures.

**Figure 8:**
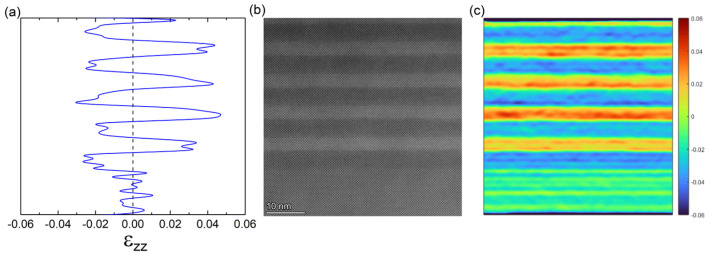
(**a**) An averaged ε_zz_ strain profile. (**b**) A HHADF-STEM image of InGaAs/InGaSb superlattice taken along [110] zone axis. (**c**) Mapping of the out-plane strain tensor ε_zz_.

Strain distribution analysis was performed using geometric phase analysis (GPA) [17] of 2048 × 2048-pixel HAADF-STEM micrographs, with the InAs homogeneous buffer layer serving as an internal reference. Figure 8c shows the mapping of the out-plane strain tensor ε_zz_, which revealed a systematic strain distribution pattern: InGaAs sub-layers exhibited negative strain due to their smaller lattice constant relative to the InAs substrate, while InGaSb sub-layers showed positive strain owing to their larger lattice constant. The averaged strain profile along the growth direction extracted from Figure 8c is drawn in Figure 8a. The periodic alternation between tensile and compressive strain regions corresponded precisely to the InGaAs and InGaSb sub-layers, respectively. The strain mapping confirmed the presence of sharp, well-defined interfaces between tensile and compressive sub-layers, corroborating the HAADF-STEM observations and validating the optimization of growth conditions.

### 3.2. Device Characterization of LWIR PIN Photodetectors

**Figure 9:**
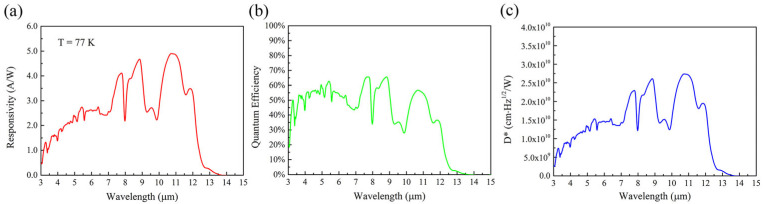
(**a**) Top illuminated responsivity of an LWIR single-element device at 77 K under 0 V applied bias. (**b**) The quantum efficiency of the single-element device. (**c**) The specific detectivity (D*) of the single-element device.

**Figure 10:**
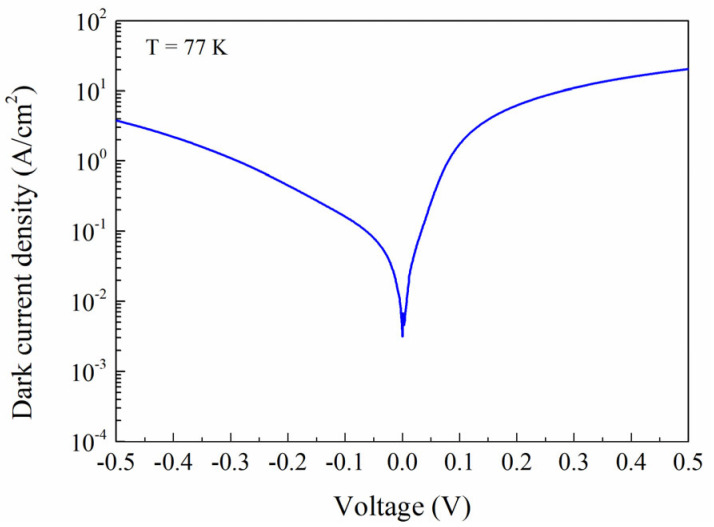
Dark current density vs. applied bias voltage characteristic of the photodetectors at 77 K.

In order to preliminarily test the performance of PIN devices, single-element PIN devices were fabricated from sample A using wet etching and standard photolithography, producing rectangular mesas ranging from 100 × 100 μm^2^ to 1000 × 1000 μm^2^ without antireflection coating or passivation treatment. Optical and electrical characterization of these long-wavelength infrared highly strained type-II superlattice (LWIR HS-T2SL) devices was performed using Fourier transform infrared (FTIR) spectroscopy. At 77 K under zero bias, the top-illuminated devices demonstrated exceptional performance metrics: a peak responsivity of 4.90 A/W, a 50% cutoff wavelength at approximately 12.1 μm (Figure 9a), and a peak quantum efficiency of 65% (Figure 9b). The specific detectivity (D*) reached 2.74 × 10^10^ cm·Hz^1/2^/W at the peak responsivity wavelength of 10.7 µm under zero bias conditions (Figure 9c). Dark current density measurements at 77 K, as shown in Figure 10, revealed consistent behavior across different mesa areas, indicating minimal surface leakage current contribution. The devices exhibited dark current densities below 5 × 10^−1^ A/cm^2^ at reverse biases less than −0.2 V, decreasing to approximately 7.96 × 10^−2^ A/cm^2^ at −50 mV.

## 4. Conclusions

This work demonstrates the successful development of highly strained InGaAs/InGaSb type-II superlattice (T2SL) structures grown on InAs substrates by molecular beam epitaxy for long-wavelength infrared detection. Through precise control of the growth parameters and comprehensive materials’ characterization, we have established the viability of this novel detector architecture. Surface morphology analysis revealed exceptional smoothness, while high-resolution X-ray diffraction and cross-sectional high-resolution transmission electron microscopy confirmed superior structural and crystalline quality, validating our growth optimization strategy. The fabricated PIN photodetector achieved a 50% cutoff wavelength of 12.1 μm and a peak responsivity of 4.90 A/W and demonstrated a dark current density of 7.96 × 10^−2^ A/cm^2^ at −50 mV when operated at 80 K. These results establish both the feasibility and reproducibility of highly strained InGaAs/InGaSb superlattice growth, providing a robust foundation for the future development of InAs-based long-wavelength infrared detection systems.

## Data Availability

Data underlying the results presented in this paper are not publicly available at this time but may be obtained from the authors upon reasonable request.
